# Topography affects not only interhemispheric asymmetry of westerly winds and thus storminess but also ocean circulation

**DOI:** 10.1073/pnas.2300613120

**Published:** 2023-02-28

**Authors:** Andreas Schmittner

**Affiliations:** ^a^College of Earth, Ocean, and Atmospheric Sciences, Oregon State University, Corvallis, OR 97331-5503

Shaw et al. (2022) (1) examined the effect of topography on the interhemispheric asymmetry of storminess, finding that topography and ocean circulation are important factors. Their paper was praised by the NSF in a press release with the title “The Southern Hemisphere is stormier than the Northern, and we finally know why.” Only that we already knew that topography affects the asymmetry of westerly winds, which are tightly related to storminess.

In 2011, we published a paper (2) demonstrating with a coupled ocean-atmosphere model that the observed asymmetry in westerly winds between the hemispheres is strongly impacted by topography. In that work, we also show that topography strongly affects ocean circulation through its impact on atmospheric water vapor transport and sea surface salinity. Unfortunately, Shaw et al. ignored our previous work, and neither the reviewers nor the editor caught that oversight. Thus, Shaw et al. not only failed to cite the relevant literature but also missed a link between topography and ocean circulation, which they have identified as the second important factor in controlling storminess because they used an atmosphere-only model. Thus, topography is likely the dominating factor in controlling the asymmetry of westerly winds and thus storminess between the hemispheres.
